# Repulsive Guidance Molecule A Suppresses Adult Neurogenesis

**DOI:** 10.1016/j.stemcr.2020.03.003

**Published:** 2020-04-02

**Authors:** Toke Jost Isaksen, Yuki Fujita, Toshihide Yamashita

**Affiliations:** 1 Department of Molecular Neuroscience, Graduate School of Medicine, Osaka University, 2-2 Yamadaoka, Suita, Osaka 565-0871, Japan; 2 WPI Immunology Frontier Research Center, Osaka University, 3-1 Yamadaoka, Suita, Osaka 565-0871, Japan; 3 Graduate School of Frontier Bioscience, Osaka University, 2-2 Yamadaoka, Suita, Osaka 565-0871, Japan; 4 Department of Neuro-Medical Science, Graduate School of Medicine, Osaka University, 2-2 Yamadaoka, Suita, Osaka 565-0871, Japan

**Keywords:** neurogenesis, hippocampus, repulsive guidance molecule, adult neural stem cells

## Abstract

Repulsive guidance molecule A (RGMa) is a glycosylphosphatidylinositol-anchored glycoprotein that exhibits repulsive neurite guidance and regulates neuronal differentiation and survival during brain development. However, the function of RGMa in the adult brain is unknown. Here, we show that RGMa is expressed in the adult hippocampus and provide evidence that RGMa signaling suppresses adult neurogenesis. Knockdown of RGMa in the dentate gyrus increased the number of surviving newborn neurons; however, these cells failed to properly migrate into the granular cell layer. *In vitro*, RGMa stimulation of adult neural stem cells suppressed neurite outgrowth of newborn neurons, which could be prevented by knockdown of the multifunctional receptor neogenin, as well as pharmacological inhibition of the downstream target Rho-associated protein kinase. These findings present a function for RGMa in the adult brain and add to the intricate molecular network that regulates adult brain plasticity.

## Highlights

• RGMa suppress survival and growth of newborn neurons in the adult dentate gyrus
• RGMa signaling depends on neogenin for the regulation of adult neurogenesis
• RGMa induces RhoA/ROCK activation in adult neuronal stem cells

## Teaser

In this article, Isaksen and colleagues show that Repulsive guidance molecule A (RGMa) suppresses adult neurogenesis. Knockdown of RGMa in the dentate gyrus increased the number of surviving newborn neurons and RGMa stimulation of adult neural stem cells suppressed neurite outgrowth, which depended on the multifunctional receptor neogenin.

## Introduction

Although the adult brain is comparatively static compared with the developing brain, substantial plastic changes in established neuronal networks still occur throughout the entire life. This includes the generation of new neurons derived from adult neural stem cells (aNSCs) (Goncalves et al., 2016). In mammals, neurogenesis occurs throughout adulthood in two defined niches: the subgranular zone (SGZ) apposed to the granular cell layer of the dentate gyrus, and the subventricular zone of the lateral ventricles (Lledo et al., 2006). In the dentate gyrus, only a fraction of newly differentiated neurons end up as mature granular neurons, integrated into the granular cell layer with functional synaptic connections (Dayer et al., 2003, Toni et al., 2007). This neuronal maturation process is regulated by intricate molecular networks, which among others control differentiation, migration, neurite growth, and synapse formation of the newborn neurons (Lledo et al., 2006). Knowledge of these regulatory pathways is important for understanding adult brain plasticity and for utilizing neural stem cells as a therapeutic tool.

Repulsive guidance molecule A (RGMa) is a glycosylphosphatidylinositol (GPI)-anchored glycoprotein that exhibits repulsive neurite guidance and regulates neuronal differentiation and survival during brain development (Matsunaga et al., 2004, Matsunaga et al., 2006, Monnier et al., 2002). The RGMa ectodomain can be shed by cleavage of its GPI anchor to generate soluble RGMa that can bind the fibronectin III domains of the multifunctional receptor neogenin (Bell et al., 2013, Tassew et al., 2012). RGMa/neogenin signaling has been linked to the inactivation of Ras via focal adhesion kinase and activation of the ras homolog gene family member A (RhoA)/Rho-associated protein kinase (ROCK) pathway, leading to cytoskeletal rearrangements, growth-cone collapse, neurite retraction, and regulation of cell death (Conrad et al., 2007, Endo and Yamashita, 2009, Siebold et al., 2017). In the adult mammalian central nervous system (CNS), RGMa is considered a negative factor for neuronal recovery in neurodegenerative disorders and injuries (Demicheva et al., 2015, Hata et al., 2006, Korecka et al., 2017); however, the functions of RGMa in the normal adult brain remain unknown. Recently, loss of neogenin was found to impair key properties of adult neurogenesis in the hippocampus, including proliferation, neurogenesis, and altered electrophysiological characteristics of newborn neurons (Sun et al., 2018). In addition, aNSC migration from the subventricular zone to the olfactory bulb is dependent on neogenin (O'Leary et al., 2015). In the adult hippocampus, neogenin is expressed in aNSCs in the SGZ and in CA3 pyramidal neurons, whereas *Rgma* mRNA has been detected in the dentate gyrus and in CA1 neurons (Sun et al., 2018, van den Heuvel et al., 2013), suggesting that RGMa/neogenin signaling could be relevant for hippocampal plasticity.

In this study, we provide evidence that RGMa is a regulator for the survival of new neurons in the dentate gyrus. *In vivo* knockdown of RGMa led to an increased number of new neurons; however, these cells seemingly failed to migrate into the granular cell layer. RGMa stimulation of cultured differentiating aNSCs caused suppression of neurogenesis and increased cell death, and the derived neurons exhibited impaired growth. Finally, *in vitro* knockdown of neogenin, as well as inhibition of ROCK signaling, prevented RGMa-induced suppression of neurite outgrowth and increased cell death.

## Results

### RGMa Can Suppress Adult Hippocampal Neurogenesis

**Figure 1:**
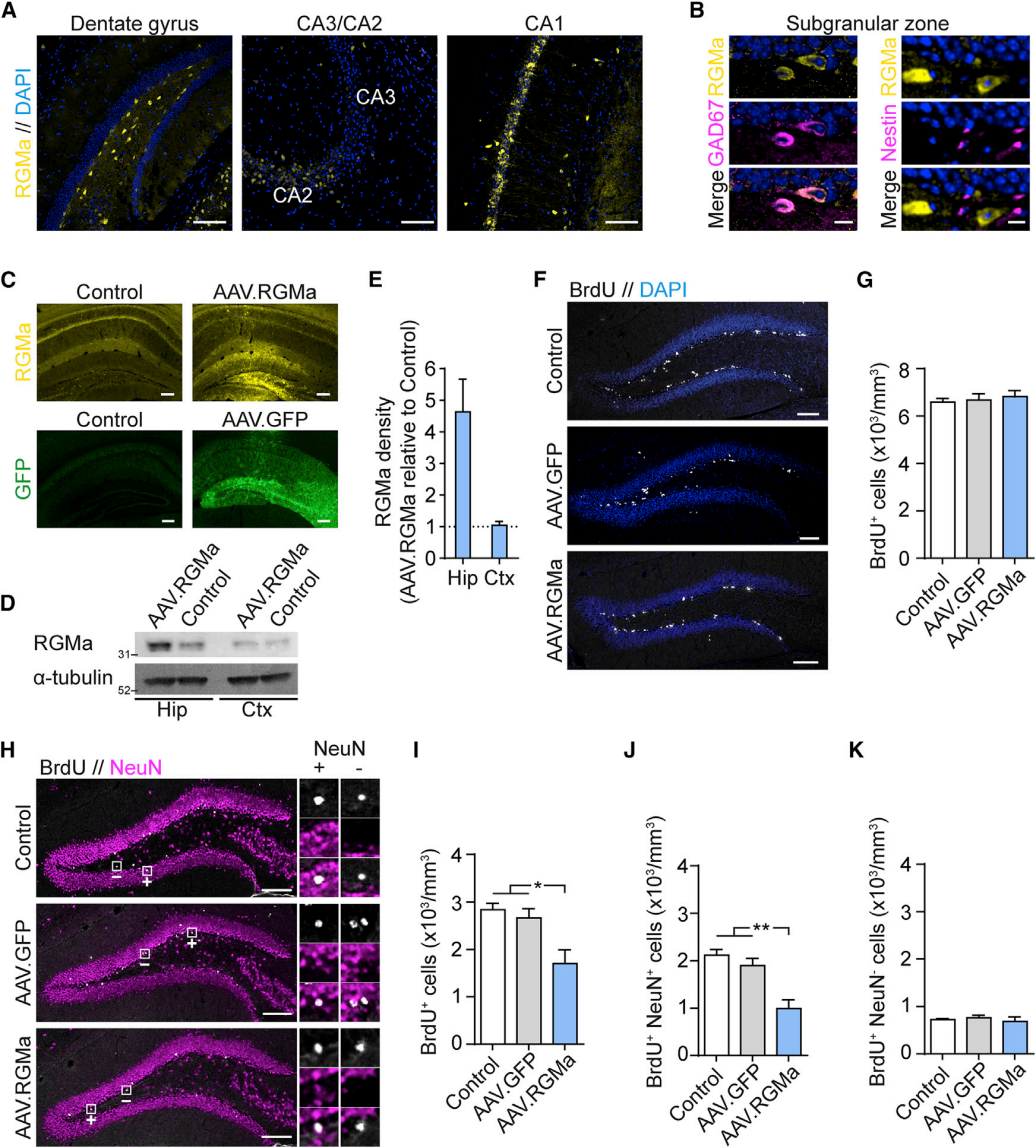
RGMa Can Suppress Adult Neurogenesis in the Hippocampus (A) IHC for RGMa in hippocampal structures. Scale bars, 100 μm. (B) IHC for RGMa together with GAD67 (left) and nestin (right) in the subgranular zone of the dentate gyrus. Nuclei were stained for DAPI (blue). Scale bars, 10 μm. (C) AAV particles were injected into the dentate gyrus, facilitating overexpression of RGMa or GFP driven by the CMV promoter. IHC for RGMa (upper images) and GFP (lower images) was performed 2 weeks after AAV infection. Scale bars, 200 μm. (D) Western blot for RGMa in the hippocampus (Hip) and cortex (Ctx) 2 weeks after AAV.RGMa infection of the dentate gyrus. Controls represent the contralateral uninfected side. (E) Relative western blot density quantification of RGMa in the hippocampus (Hip) and cortex (Ctx) presented as the value of the AAV.RGMa-infected side relative to that of the control side (mean ± SEM; n = 3 mice). (F and G) Noninfected control and AAV-infected mice were administered BrdU for four consecutive days and sacrificed on the fifth day. The number of BrdU^+^ cells within the dentate gyrus was analyzed by IHC. Scale bars, 100 μm. Mean ± SEM; n = 5, 5, 6 mice; one-way ANOVA followed by Tukey's multiple comparisons test. (H–K) Control and AAV-infected mice were administered BrdU for 4 consecutive days and sacrificed 4 weeks later. The numbers of BrdU^+^ cells (I), BrdU^+^ NeuN^+^ cells (J), and BrdU^+^ NeuN^−^ cells (K) within the dentate gyrus were analyzed by IHC. Enlarged images (H, right) demonstrate examples of BrdU^+^ NeuN^+^ cells and BrdU^+^ NeuN^−^ cells within the dentate gyrus (upper, BrdU; middle, NeuN; lower, merge). Mean ± SEM; n = 5 mice; one-way ANOVA followed by Tukey's multiple comparisons test: ^∗^p < 0.05, ^∗∗^p < 0.01. Scale bars, 100 μm.

To investigate the activity of RGMa in the adult brain, we first assessed RGMa expression by immunohistochemistry (IHC) in 8-week-old mice. In the hippocampus a distinct RGMa pattern was observed, with prominent RGMa staining in the dentate gyrus as well as in the CA2/CA1 pyramidal cell layer (Figure 1A). Comparable hippocampal RGMa expression was also detected by *in situ* hybridization for *Rgma* mRNA in adult mice (Figure S1) (Lein et al., 2007, van den Heuvel et al., 2013). In the dentate gyrus, RGMa staining was observed primarily in a seemingly distinct group of hilus cells. Double staining with cell markers revealed that most RGMa-expressing hilus cells were positive for the GABAergic interneuron marker GAD67, with 32% ± 17% of hilar interneurons expressing RGMa (Figure 1B, left). Furthermore, RGMa-positive cells were frequently observed in close proximity to nestin-positive aNSCs in the SGZ (Figure 1B, right).

To first test whether hippocampal aNSCs respond to RGMa signaling, we induced adeno-associated virus (AAV)-mediated overexpression of RGMa in the dentate gyrus. For this, full-length RGMa was amplified from adult hippocampal cDNA and cloned into the pAAV-MCS vector (Figure S2A). Viral-infected 293 cells exhibited distinctive RGMa processing and membrane staining (Figures S2B and S2C). Viral particles were stereotaxically injected into the dentate gyrus, giving rise to an apparent overexpression of RGMa, or green fluorescent protein (GFP) as a viral control, throughout the structure (Figures 1C and S2D). Western blot analysis of the 33-kDa C-terminal processed form of RGMa (Tassew et al., 2012) showed a 462% ± 104% increase in RGMa in the infected hippocampus compared with the noninfected control side (Figures 1D and 1E). No change in RGMa expression was observed in the surrounding cortical tissue (Figures 1D and 1E).

Two weeks after AAV infection, dividing cells were labeled with injection of bromodeoxyuridine (BrdU) for 4 days. To determine whether RGMa overexpression could affect proliferation of aNSCs, we sacrificed mice on the fifth day and assessed BrdU incorporation within the subgranular zone by IHC (Figure 1F). No effect was observed on the number of BrdU-positive (BrdU^+^) cells among noninfected control, AAV.GFP-infected, and AAV.RGMa-infected mice (p = 0.7633) (Figure 1G).

Next, to test whether RGMa overexpression could affect fate and survival of proliferating aNSCs, we performed IHC for BrdU and the neuronal marker NeuN 4 weeks after the 4-day BrdU pulse (Figure 1H). At this time point, most BrdU-labeled aNSCs would be expected to have differentiated and either been integrated as mature cells including mature NeuN^+^ neurons or suffered cell death via apoptosis. Interestingly, the number of surviving BrdU^+^ cells was significantly decreased in AAV.RGMa-infected mice but not in AAV.GFP-infected mice, compared with control mice (p = 0.0146) (Figure 1I). To determine the affected cell types, we analyzed the numbers of newborn neurons (BrdU^+^ NeuN^+^) and new non-neuronal cells (BrdU^+^ NeuN^−^) (Figure 1H, small images). The number of BrdU^+^ NeuN^+^ cells in AAV.RGMa-infected mice was significantly lower compared with AAV.GFP-infected and control mice (p = 0.0013) (Figure 1J). Conversely, no changes in the numbers of BrdU^+^ NeuN^−^ cells were observed between the three groups of mice (p = 0.9256) (Figure 1K). These results suggest that RGMa can suppress neurogenesis but neither gliogenesis nor proliferation.

### Knockdown of RGMa Enhances Survival but Disrupts Migration of Newborn Neurons

**Figure 2:**
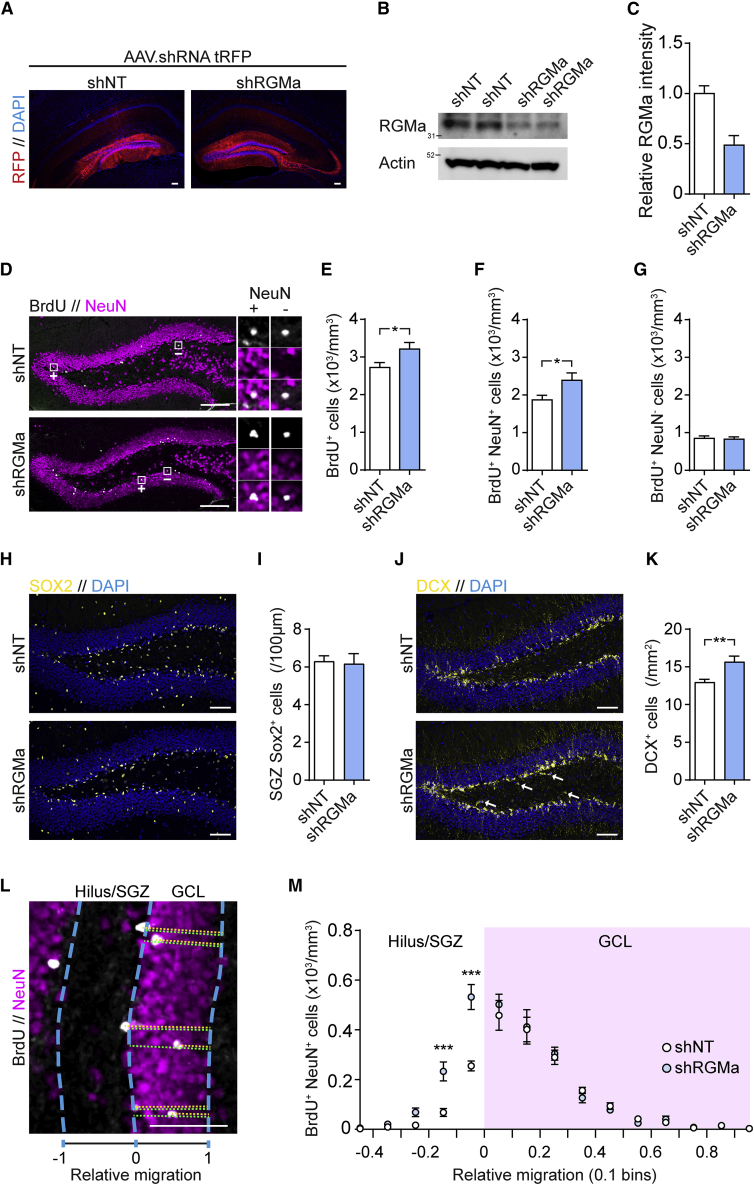
Knockdown of RGMa Increases the Number of New Neurons (A) AAV.shRNA tRFP particles were injected into each dentate gyrus to facilitate the shRNA-mediated knockdown of RGMa (shRGMa) or no target (shNT). Scale bars, 100 μm. (B) Western blot for RGMa in the dentate gyrus 2 weeks after AAV infection with shNT and shRGMa in two mice. (C) Relative western blot density quantification of RGMa in the dentate gyrus 2 weeks after AAV infection. Mean ± SEM; n = 6 mice; unpaired Student's t test. (D–G) AAV-infected mice were administered BrdU for 4 consecutive days and sacrificed 4 weeks later. The numbers of BrdU^+^ cells (E), BrdU^+^ NeuN^+^ cells (F), and BrdU^+^ NeuN^−^ cells (G) within the dentate gyrus were analyzed by IHC. Mean ± SEM; n = 7 mice; unpaired Student's t test: ^∗^p < 0.05. Scale bars, 100 μm. (H and I) AAV-infected mice were stained for the stem cell marker SOX2 and the number of SOX2^+^ cells along the SGZ was counted. Mean ± SEM; n = 5 mice; unpaired Student's t test. Scale bars, 100 μm. (J and K) AAV-infected mice were stained for the immature neuronal marker DCX and the number of DCX^+^ neurons was determined and normalized to the area of the granular cell layer (GCL). Mean ± SEM; n = 6 mice; unpaired Student's t test. Scale bars, 100 μm. (L and M) Frequency analysis of relative migration of BrdU^+^ NeuN^+^ cells into the granular cell layer (GCL). Mean ± SEM; n = 7 mice; two-way ANOVA followed by Sidak's multiple comparisons test: ^∗∗∗^p < 0.001. Scale bar, 50 μm.

Loss of RGMa is embryonic lethal (Niederkofler et al., 2004); however, previous animal studies have successfully used viral vectors for knockdown of RGMa (Zhang et al., 2018). Therefore, to test whether endogenous RGMa also regulates adult hippocampal neurogenesis, we delivered RGMa (shRGMa) or nontargeted (shNT) short hairpin RNA (shRNA) to the dentate gyrus by AAV particles (Figures 2A and S3A). Two weeks after the viral infection, western blot analysis of the 33-kDa C-terminal processed form of RGMa in the dentate gyrus showed a 56% ± 6% reduction after shRGMa infection compared with shNT infection (Figures 2B and 2C). A similar decrease in *Rgma* mRNA after shRGMa infection was observed in the dentate gyrus by qPCR analysis, whereas *Neogenin* mRNA expression was not affected by shRGMa infection (p < 0.0001 and p = 0.6483, respectively) (Figures S3B and S3C).

Next, IHC for BrdU and NeuN was performed 4 weeks after a 4-day BrdU pulse in shRNA AAV-infected animals (Figure 2D). Compared with shNT-infected mice, shRGMa-infected mice exhibited a small but significantly higher number of BrdU^+^ cells in the dentate gyrus (p = 0.0369) (Figure 2E). Upon analyzing BrdU-positive cells for NeuN-positive co-staining, the number of newborn neurons (BrdU^+^ NeuN^+^) was significantly increased in shRGMa-infected mice (p = 0.0133) (Figure 2F). No difference was observed for new non-neuronal cells (BrdU^+^ NeuN^−^) between shRGMa and shNT infection (p = 0.8194) (Figure 2G). To test whether the effect of RGM knockdown was caused by the change in progenitor cells numbers, we performed IHC for the stem cell marker SOX2 in shRNA-infected animals (Figure 2H). No difference in number of SOX2^+^ cells along the SGZ between shRGMa and shNT infection was observed (p = 0.7514) (Figure 2I). As RGMa regulates neuronal growth, differentiation, and survival during early neuronal development in the developing brain (Matsunaga et al., 2004, Matsunaga et al., 2006, Monnier et al., 2002), we next checked the effect of RGMa on developing newborn neurons using the immature neuronal marker doublecortin (DCX) (Figure 2J). ShRGMa infection caused a significant increase of DCX^+^ cells in the dentate gyrus compared with shNT infection (p = 0.0088) (Figure 2K).

Interestingly, some DCX^+^ neurons were observed in the hilar region of the dentate gyrus after shRGMa infection (Figure 2J, white arrows). This was rarely observed after shTN infection. Newborn neurons normally migrate tangentially along the SGZ, then radially into the granular cell layer, during their maturation process (Sun et al., 2015). As this migration process is important for integration and survival, we speculated that RGMa might affect neuronal migration. To test this, we measured the relative migration of BrdU^+^ NeuN^+^ cells (newborn mature neurons) into the granular cell layer (Figure 2L) and assessed it by frequency analysis (Figure 2M). A notable change in the distribution of BrdU^+^ NeuN^+^ cells was observed outside the granular cell layer, with a significantly higher number of cells located in the hilus/SGZ area (−0.2, −0.1 bins) after shRGMa compared with shNT infection (p = 0.0002 and <0.0001, respectively). However, no change was observed in the number of cells migrating into the granular cell layer (≥0 bins) between the two shRNA infections, suggesting that RGMa is not essential for the radial migration itself but is potentially involved in eliminating newborn neurons that fail to migrate into the granular cell layer.

### RGMa Suppresses Neurogenesis *In Vitro*

**Figure 3:**
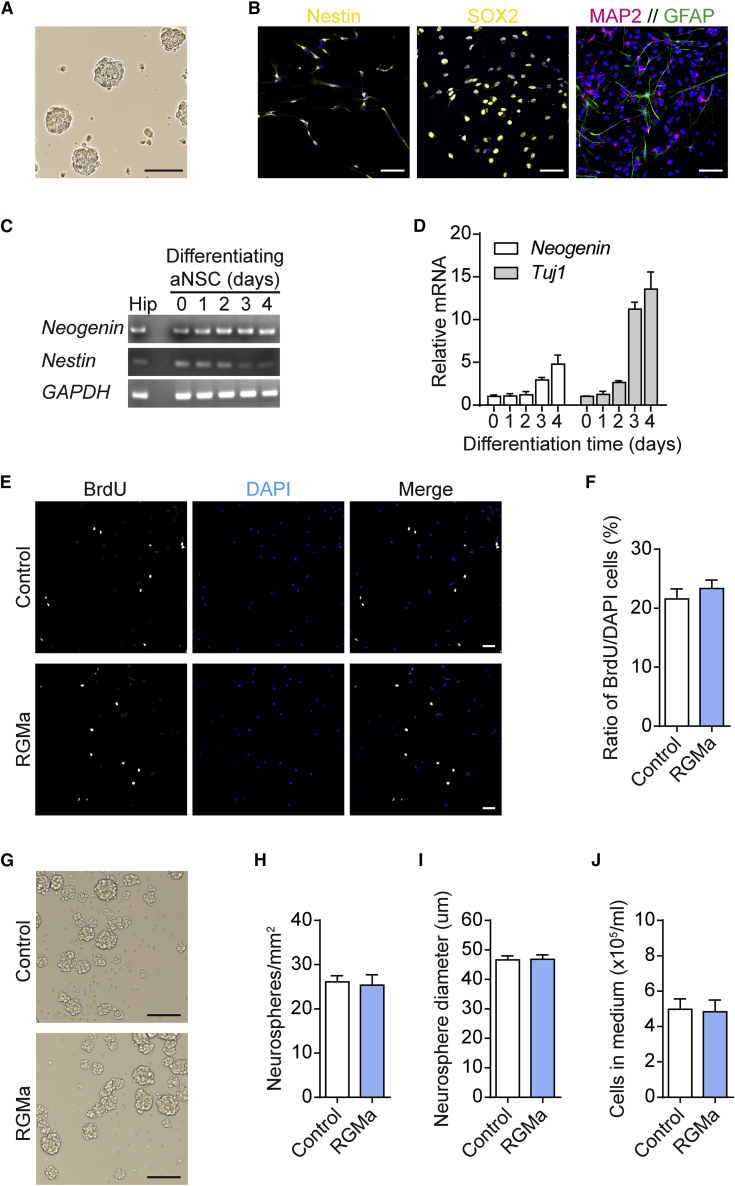
RGMa Does Not Affect the Proliferation of aNSCs *In Vitro* (A) aNSCs cultured *in vitro* as neurospheres. Scale bar, 100 μm. (B) Cultured aNSCs express the neural progenitor markers nestin and SOX2 under proliferating conditions and can be induced to differentiate into MAP2^+^ neurons and GFAP^+^ astrocytes. Nuclei are stained with DAPI (blue). Scale bars, 50 μm. (C and D) Expression of *Neogenin* probed by two-step RT-PCR (C) and qPCR (D) in the adult hippocampus and in cultured aNSCs during 5 days of differentiation. Mean ± SEM; n = 3 independent experiments. (E) aNSCs cultured under proliferating conditions and stimulated with RGMa incorporated BrdU in cell proliferation analyses. Scale bars, 50 μm. (F) Quantitative analysis of BrdU incorporation in relation to the total number of DAPI-stained cells. Mean ± SEM; n = 4 independent experiments; unpaired Student's t test. (G–J) aNSCs were seeded into 24-well culture plates at 1 × 10^5^ cells/mL in proliferation medium and stimulated with RGMa. After 48 h, the numbers of formed neurospheres (H), average neurosphere diameters (I), and cell concentrations after dissociation of formed neurospheres (J) were assessed. Mean ± SEM; n = 6, 3 independent experiments; unpaired Student's t test. Scale bars, 100 μm.

To further investigate the effect of RGMa under more controllable conditions, we isolated aNSCs from the dentate gyrus of 8-week-old mice and cultured them *in vitro* as neurospheres (Figure 3A). These proliferating aNSCs were positive for the NSC markers nestin (97%) and SOX2 (93%) and could be induced to differentiate into microtubule-associated protein 2 (MAP2)-positive neurons (MAP2^+^) and glial fibrillary acidic protein (GFAP)-positive astrocytes (GFAP^+^) (Figure 3B). Consistent with previous findings (Sun et al., 2018), cultured aNSCs exhibited *Neogenin* mRNA expression (Figure 3C), which increased up to 6-fold after 5 days of differentiation (Figure 3D).

First, to assess stem cell proliferation in the presence of RGMa, aNSCs were seeded into laminin-coated chamber wells. Two hours later, cells were stimulated with recombinant soluble mouse RGMa (1 μg/mL) for 20 h and then labeled with a 6-h BrdU pulse (Figure 3E). Quantification of BrdU-positive cells relative to the total number of DAPI-stained cells demonstrated no significant effect of RGMa on cell proliferation (p = 0.4542) (Figure 3F). Similarly, neurosphere proliferation assay showed no effect of RGMa stimulation on the number of formed neurospheres, average neurosphere diameter, or total number of cells after neurosphere dissociation (Figures 3G–3J). Thus, consistent with the *in vivo* results (Figure 1G), RGMa stimulation did not affect aNSC proliferation *in vitro*.

**Figure 4:**
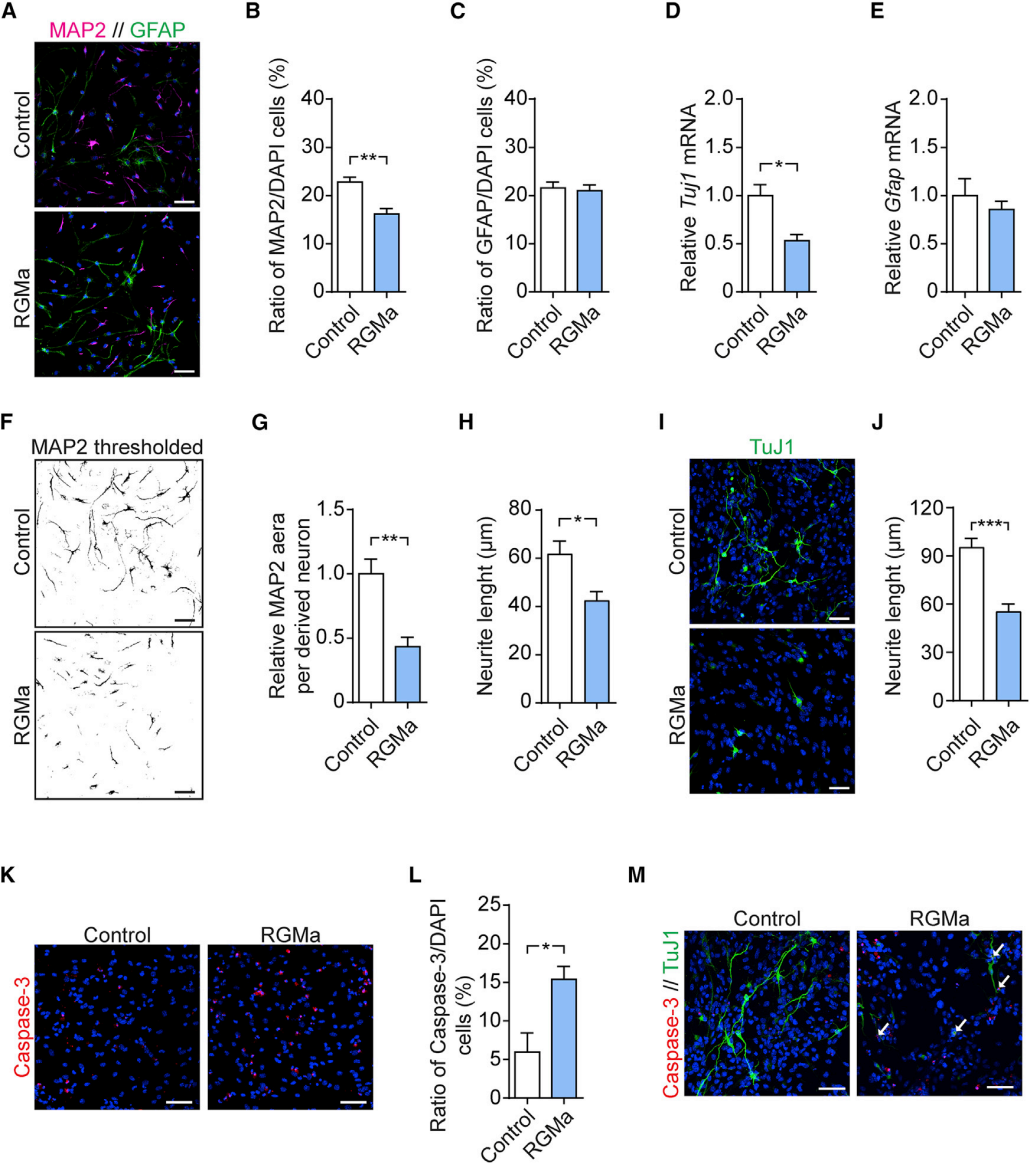
RGMa Suppresses Neurogenesis *In Vitro* (A–C) Differentiating aNSCs were stimulated with RGMa for 5 days and the numbers of MAP2^+^ (B) and GFAP^+^ (C) cells in relation to the total number of DAPI-stained cells (blue) was quantified by immunocytochemistry. mean ± SEM; n = 8, 5 independent experiments; unpaired Student's t test: ^∗∗^p < 0.01. Scale bars, 50 μm. (D and E) qPCR analyses of *Tuj1* (D) and *Gfap* (E) mRNA levels in differentiating aNSCs stimulated with RGMa. Mean ± SEM; n = 3 independent experiments; unpaired Student's t test: ^∗^p < 0.05. (F and G) The area of differentiated MAP2^+^ neurons was analyzed by an automated threshold area analysis (F), and the average MAP2 area per neuron was calculated (G). Mean ± SEM (normalized to control); n = 8, 5 independent experiments; unpaired Student's t test: ^∗∗^p < 0.01. Scale bars, 50 μm. (H) Neurite length was measured in MAP2^+^ neurons by neurite tracing. Mean ± SEM; n = 8, 5 independent experiments; unpaired Student's t test. (I and J) Differentiating aNSCs were stimulated with RGMa for 6 days and the neurite length in TuJ1^+^ neurons was determined by neurite tracing. Mean ± SEM; n = 5 independent experiments; unpaired Student's t test. Scale bars, 50 μm. (K and L) Cell death in differentiating aNSCs stimulated with RGMa was evaluated by immunocytochemistry for cleaved caspase-3 (N), and the ratio of apoptotic cleaved caspase-3-positive cells in relation to the total number of DAPI-stained cells (blue) was determined (O). Mean ± SEM; n = 4 independent experiments; unpaired Student's t test: ^∗^p < 0.05. Scale bars, 50 μm. (M) Double staining for cleaved caspase-3 and TuJ1 in differentiating aNSCs stimulated with RGMa. Co-localization was often observed in cells stimulated with RGMa (white arrows). Scale bars, 50 μm.

Next, to evaluate the effect of RGMa on neurogenesis, we seeded aNSCs into laminin-coated chamber wells. The following day, cell differentiation was induced and RGMa (1 μg/mL) was added to the cells. Following 5 days of differentiation with RGMa stimulation, cells were fixed and stained for MAP2 and GFAP (Figure 4A). Quantification of MAP2^+^ and GFAP^+^ cells showed a significant suppressing effect of RGMa stimulation on neurogenesis, with a decrease in the ratio of MAP2^+^ neurons compared with unstimulated cells (p = 0.0014) (Figure 4B). No effect of RGMa on the generation of GFAP^+^ astrocytes was observed (p = 0.7610) (Figure 4C). This observation was further verified by qPCR for β-tubulin III (*Tuj1*) and *Gfap* expression in differentiating aNSCs. RGMa-stimulated cells exhibited a significant decrease in *Tuj1* mRNA compared with control cells (p = 0.0237) (Figure 4D), whereas no difference was observed for *Gfap* mRNA (p = 0.5002) (Figure 4E). Furthermore, aNSCs stimulated with RGMa exhibited a reduced MAP2 area per neuron (p = 0.0043) (Figures 4F and 4G), suggesting that the neurite growth of neurons was suppressed by RGMa. To confirm this, we determined neurite length by neurite tracing on derived MAP2^+^ neurons. Consistent with the reduced MAP2 area, RGMa-stimulated neurons exhibited a significant reduction in neurite length compared with unstimulated cells (p = 0.0248) (Figure 4H). As RGMa primarily is regarded as an axonal growth regulator (Matsunaga et al., 2006), RGMa-stimulated aNSCs were stained for the axonal specific marker TuJ1 (Figure 4I). Neurite tracing of TuJ1^+^-derived neurons showed a pronounced inhibitory effect of RGMa stimulation on neurite length compared with unstimulated control cells (p = 0.0005) (Figure 4J).

RGMa stimulation during aNSC differentiation also induced cell death, with a significantly higher ratio of active caspase-3-positive cells after RGMa stimulation compared with unstimulated control cells (p = 0.0199) (Figures 4K and 4L). These apoptotic cells stimulated with RGMa frequently showed co-localization of active caspase-3 and TuJ1 during the early phase of apoptosis before breakdown of the cell structure (Figure 4M, white arrows). This co-localization was rarely observed in unstimulated apoptotic cells (Figure 4M). This could suggest that RGMa primarily affect survival of newborn neurons, which would be consistent with the observation that RGMa stimulation only decreased the ratio of MAP2^+^ neurons but not the ratio of GFAP^+^ astrocytes (Figures 4B and 4C). However, these data do not rule out the possibility that non-neuronal cells also can undergo cell death following RGMa stimulation.

### RGMa Effects Depend on Neogenin and RhoA/ROCK Activity

**Figure 5:**
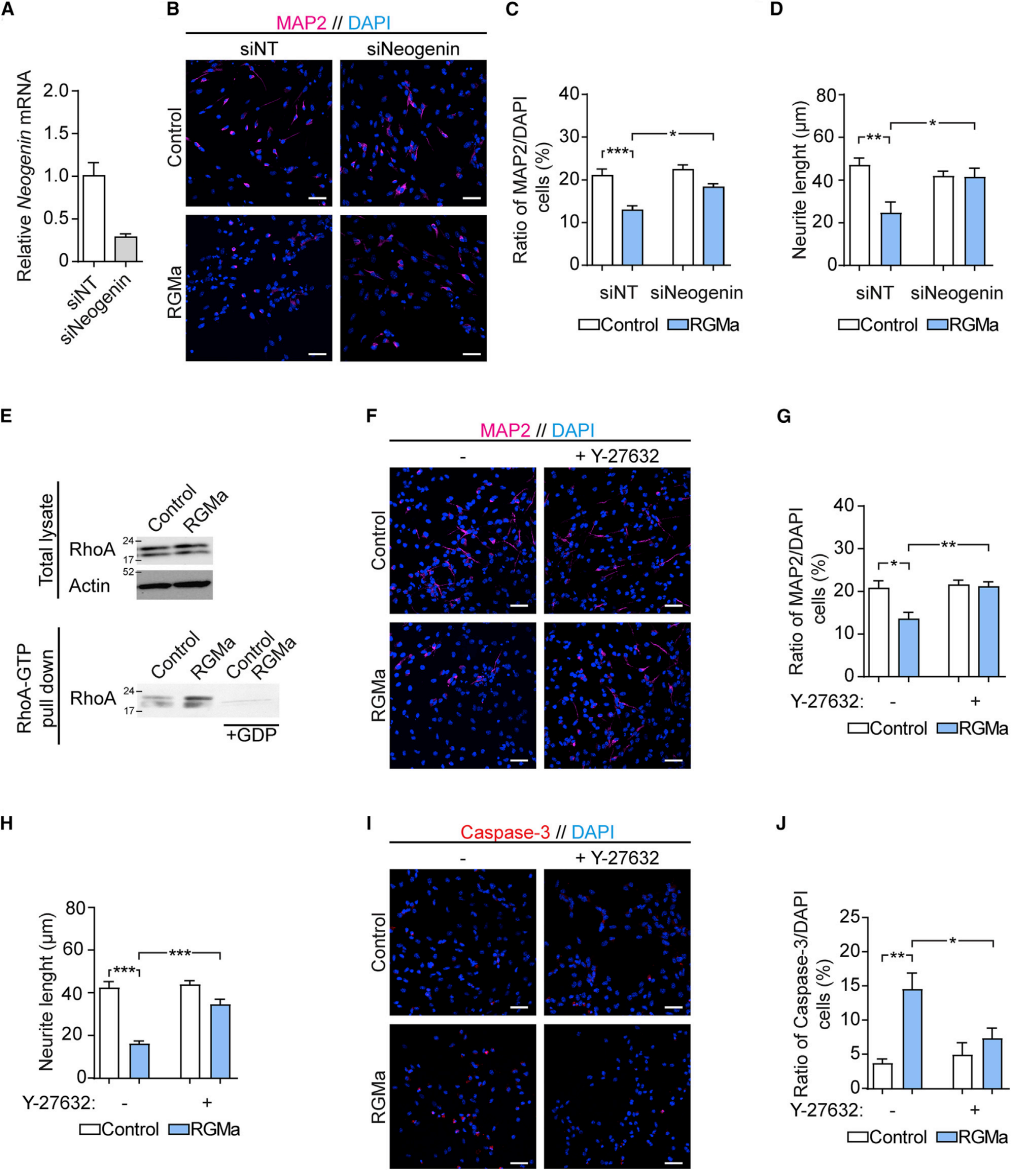
RGMa Suppression Depends on Neogenin and Activates the RhoA/ROCK Pathway (A) Cultured aNSCs were transfected with siRNA against neogenin (siNeo) or no target (siNT). After 36 h, neogenin mRNA levels were analyzed by qPCR. Mean ± SEM; n = 5 independent experiments; unpaired Student's t test. (B–D) After 36 h following siRNA transfection, aNSCs were induced to differentiate and stimulated with RGMa for 5 days. (C) The number of MAP2^+^ cells in relation to the total number of DAPI-stained cells was quantified by immunocytochemistry. Mean ± SEM; n = 6 independent experiments. (D) Neurite length of differentiated MAP2^+^ neurons was analyzed by neurite tracing. Mean ± SEM; n = 6 independent experiments; one-way ANOVA followed by Tukey's multiple comparisons test: ^∗^p < 0.05, ^∗∗^p < 0.01. Scale bars, 50 μm. (E) Total RhoA (total lysate) and active RhoA (RhoA-GTP pulled down by Rhotekin-RBD agarose beads) in aNSCs after stimulation with RGMa was analyzed by western blot. GDP was added during the pull-down as a control for the RhoA-GTP pull-down specificity. (F–H) Differentiating aNSCs was stimulated with RGMa and the ROCK inhibitor Y-27632 for 5 days, and the number of MAP2^+^ cells (G) in relation to the total number of DAPI-stained cells was quantified by immunocytochemistry. Mean ± SEM; n = 6 independent experiments; one-way ANOVA followed by Tukey's multiple comparisons test. (H) Neurite length of differentiated MAP2^+^ neurons was analyzed by neurite tracing. Mean ± SEM; n = 6 independent experiments; one-way ANOVA followed by Tukey's multiple comparisons test. ^∗^p < 0.05, ^∗∗^p < 0.01, ^∗∗∗^p < 0.001. Scale bars, 50 μm. (I and J) Cell death in differentiating aNSCs stimulated with RGMa and Y-27632 was evaluated by immunocytochemistry for cleaved caspase-3 (I), and the ratio of apoptotic cleaved caspase-3 positive cells in relation to the total number of DAPI-stained cells was determined (J). Mean ± SEM; n = 5 independent experiments; one-way ANOVA followed by Tukey's multiple comparisons test: ^∗^p < 0.05, ^∗∗^p < 0.01. Scale bars, 50 μm.

Neogenin is the main receptor for RGMa and exhibits its inhibitory effect on neurons in the developing brain (Rajagopalan et al., 2004). To test whether the effect of RGMa on adult neurogenesis is dependent on neogenin, we transfected aNSCs with neogenin-targeted small interfering RNA (siRNA) Neogenin (siNeogenin), resulting in a 78% ± 6% decrease in *Neogenin* mRNA compared with cells transfected with nontargeted siRNA (siNT) (Figure 5A). The effect of RGMa stimulation on differentiating siRNA-transfected aNSCs was then examined by MAP2 staining (Figure 5B). Transfection with siNT did not prevent neuronal suppression by RGMa; however, siNeogenin-transfected cells stimulated with RGMa showed a significant increase in the number of MAP2^+^ neurons compared with siNT-transfected cells stimulated with RGMa (p = 0.0236) (Figure 5C). Similarly, neogenin knockdown largely prevented neurite growth suppression by RGMa, with a significant increase in neurite length after RGMa stimulation with siNeogenin transfection compared with RGMa stimulation with siNT transfection (p = 0.0324) (Figure 5D). This suggests that RGMa signaling mainly depends on neogenin in aNSCs.

A key downstream pathway of RGMa signaling is the activation of RhoA, which in turn activates ROCK, leading to cytoskeletal rearrangements and inhibition of neurite growth (Conrad et al., 2007). To test whether RGMa stimulation of aNSCs leads to RhoA activation, we performed pull-down of activated RhoA (RhoA-GTP) by Rhotekin-Rho binding domain (RBD) beads. Subsequent western blot analyses showed an increase in active RhoA, but no change in total RhoA, in differentiating aNSCs stimulated with RGMa compared with unstimulated control cells (Figure 5E). Next, neuronal differentiation of aNSCs by RGMa was tested together with the selective ROCK inhibitor Y-27632 (Figure 5F). Quantification of MAP2^+^ neurons after 5 days of differentiation showed that Y-27632 prevented RGMa-induced suppression of neurogenesis (p = 0.0079) (Figure 5G). Y-27632 treatment also prevented RGMa suppression of neurite length (p < 0.001) (Figure 5H). Finally, caspase-3 staining (Figure 5I) showed that ROCK inhibition significantly reduced cell death in differentiating aNSCs stimulated with RGMa (p = 0.0417) (Figure 5J). Thus, activation of the downstream RhoA/ROCK pathway is central in RGMa-induced suppression of neurogenesis.

## Discussion

The development of functional and integrated granule neurons derived from aNSCs is strictly controlled in the adult hippocampus, and only a fraction of neuronally differentiating aNSCs survive this selection process (Dayer et al., 2003). After fate determination, newborn neurons first migrate tangentially and then radially from the SGZ into the granular cell layer (Sun et al., 2015). At the same time, neurite outgrowth and axonal/dendritic formation begins and synaptic connections are formed (Goncalves et al., 2016, Lledo et al., 2006, Toni et al., 2007). Our data provide evidence for a role of RGMa in the survival of newborn neurons in the hippocampus. Knockdown of RGMa did not affect the SOX2^+^ stem cell population but increased the number of both newborn immature (DCX^+^) and mature (BrdU^+^ NeuN^+^) neurons. However, some of these newborn neurons failed to properly migrate into the granular cell layer as a result of RGMa knockdown. Furthermore, differentiating newborn neurons exhibited impaired neurite growth when stimulated with soluble RGMa *in vitro*. Given the largely hilar expression of RGMa, it is possible that cleaved soluble RGMa peptides (Tassew et al., 2012) released from nearby inhibitory neurons suppresses growth and survival of newborn neurons that fail to migrate into the granular cell layer. In support of this, subventricular zone-derived aNSCs depend on neogenin and a netrin-1 gradient for proper migration into the olfactory bulb from the rostral migratory stream (O'Leary et al., 2015). Netrin-1/neogenin signaling demonstrates chemoattractant activity for developing neurons and has been shown to act competitively to RGMa/neogenin repulsive signaling (Serafini et al., 1996, Wilson and Key, 2006). In the hilus of the dentate gyrus, RGMa expression was observed primarily in GAD67-positive inhibitory neurons. These inhibitory neurons have previously been implicated as vital regulators of adult neurogenesis. For instance, secretion of the extracellular glycoprotein reelin by hilar inhibitory neurons is important for proper migration of newborn neurons into the granular cell layer (Gong et al., 2007), and GABAergic synaptic inputs to new neurons are decisive for activity-dependent survival and neuronal maturation (Heigele et al., 2016). Taken together, our data and reports by others provide strong evidence that inhibitory neurons of the dentate gyrus are crucial for several aspects of neurogenesis in the adult brain.

RGMa expression was also observed in the CA1/2 region; however, its function here remains unclear. The inverse expression of neogenin in CA3 pyramidal neurons (Sun et al., 2018) and RGMa in CA1 could suggest a role for RGMa in affecting the synaptic plasticity of Schaffer collateral connections between CA3 and CA1.

The main receptor for RGMa in the developing brain is neogenin (Rajagopalan et al., 2004), and RGMa/neogenin signaling has been implicated in several key aspects of neuronal development, including neurite growth and guidance, differentiation, and survival (Hata et al., 2006, Matsunaga et al., 2004, Matsunaga et al., 2006, Monnier et al., 2002). We found that neogenin knockdown in cultured aNSCs prevented RGMa-induced suppression of neurogenesis and neurite growth, demonstrating that RGMa also depends on neogenin for the regulation of adult neurogenesis. Neogenin is expressed in many neuronal and glial precursors throughout the developing brain and in SGZ and subventricular-zone aNSC populations in the adult brain (Fitzgerald et al., 2006a, Fitzgerald et al., 2006b, Sun et al., 2018). Loss of neogenin in SGZ aNSCs impairs proliferation and neurogenesis (Sun et al., 2018) which, given our findings, suggests that other neogenin ligands act antagonistically and dissimilarly to RGMa. Besides RGMa, known neogenin ligands consist of netrins and bone morphogenetic proteins (BMPs) (Hagihara et al., 2011, Keino-Masu et al., 1996). Neogenin can furthermore act as a dependence receptor (Matsunaga et al., 2004). BMPs are recognized as critical regulators of stem cell proliferation and maintenance in a wide variety of niches (Morrison and Spradling, 2008), including in the adult hippocampus where BMP signaling inhibits aNSC proliferation while maintaining stem cell activity that promotes continuous neurogenesis (Bonaguidi et al., 2008, Mira et al., 2010). While netrin-1 is important for aNSC migration from the subventricular zone (O'Leary et al., 2015), the function of netrins in hippocampal neurogenesis still remains to be elucidated. Nevertheless, epileptic seizures have been reported to induce hilar netrin-1 expression, which is associated with an abnormal migration of newborn neurons into the hilus (Yang et al., 2008). RGMa has also been shown to bind other receptors, including as a cofactor for BMP signaling (Siebold et al., 2017). Thus, while neogenin knockdown had a significant abrogating effect on RGMa suppression, it still remains to be elucidated whether RGMa also can signal through other receptors in aNSCs.

RGMa stimulation led to a marked increase in active RhoA, and inhibition of its downstream target ROCK prevented RGMa suppression of neurite growth and cell death. RGMa-mediated activation of the RhoA/ROCK pathway has indeed previously been reported as a major intracellular pathway for RGMa effects on neurons (Conrad et al., 2007, Hata et al., 2006, Hata et al., 2009). ROCK is a key effector of the Rho family of guanosine triphosphatases (GTPases) and acts as an important hub for control of actin-cytoskeleton rearrangements in most mammalian cells, including in developing neurons where ROCK activity is a critical factor for regulation of axonal outgrowth (Bito et al., 2000, Ishizaki et al., 1996, Riento and Ridley, 2003). It is, therefore, no surprise that both our data and reports by others provide evidence that RhoA and ROCK activity is also important for adult neurogenesis. For instance, loss of the Rho GTPase regulator oligophrenin-1, which leads to ROCK overactivation, strongly suppresses adult neurogenesis but not aNSC proliferation in the hippocampus (Allegra et al., 2017, Khelfaoui et al., 2009). Pharmacological inhibition of ROCK activity can restore adult neurogenesis in oligophrenin-1 knockout mice and also increase the number of surviving newborn neurons in wild-type mice (Allegra et al., 2017, Christie et al., 2013). Furthermore, aNSCs respond biomechanically to extracellular matrix stiffness through activation of RhoA and Cdc42, which in turn suppress neurogenesis (Keung et al., 2011), and ROCK activity decreases in parallel with the development of neuronal-like morphology during *in vitro* neurogenesis (Compagnucci et al., 2016).

Our data suggest that some newborn neurons undergo cell death when stimulated with RGMa. However, exactly which population of precursor neurons is primarily affected and whether other cell types are also undergoing cell death due to RGMa stimulation remains to be confirmed. Neogeinin can act a dependence receptor (Matsunaga et al., 2004), which induces cell death when no neogenin ligand is present. However, given that ROCK inhibition alleviated RGMa-induced cell death in this study, we suggest that the increased cell death could be a consequence of impaired growth and not related to the dependence function of neogenin. In support of this, activation of RhoA and ROCK in developing neurons and after a neuronal injury has also been linked to regulation of apoptosis (Dergham et al., 2002, Kobayashi et al., 2004, Sanno et al., 2010).

Following ischemic stroke and other types of brain injuries, increased proliferation rates of aNSCs in the SGZ, as well as in the subventricular zone, have been reported (Liu et al., 1998, Nakatomi et al., 2002). Furthermore, endogenous aNSCs can migrate from their original niches toward the affected injured area. Here a low number of these migrating aNSCs survive and differentiate into neurons and glial cells that can integrate into the injured area, presumably replacing lost cells and supporting neuronal recovery (Arvidsson et al., 2002, Nakatomi et al., 2002, Yamashita et al., 2006). RGMa has been extensively studied for its potential inhibitory role in neuronal recovery in neurodegenerative disorders and after CNS injuries (Demicheva et al., 2015, Hata et al., 2006, Korecka et al., 2017, Wang et al., 2018). Following neuronal damage, RGMa expression increases locally at the lesion area (Schwab et al., 2005), and inhibition of RGMa activity has led to improved functional recovery (Demicheva et al., 2015, Hata et al., 2006). Our data propose that RGMa negatively regulates the growth and survival of newborn neurons in the adult brain. Thus, it is possible that local RGMa inhibition at the lesion site following brain injury can promote survival and growth of newborn neurons derived either from migrating endogenous aNSCs or therapeutically delivered exogenous stem cells.

## Experimental Procedures

### Animals

All experiments were performed in 8- to 14-week-old C57BL/6NCrSlc mice (Japan SLC). All experimental animal protocols were approved and performed according to the institutional regulations of Osaka University.

### Isolation and Passaging of aNSCs

aNSC isolation was adapted from Guo et al. (2012). For each preparation, a single mouse brain was sliced into 500-μm coronal sections. The dentate gyrus was surgically collected from sections spanning the hippocampus and minced in ice-cold Hank’s balanced salt solution containing 30 mM glucose, 2 mM HEPES, and 26 mM NaHCO_3_. Digestion was performed in 0.05% trypsin-EDTA (T4174, Sigma-Aldrich) for 20 min and stopped by adding an equal volume of trypsin inhibitor (T6522, Sigma-Aldrich) in phosphate-buffered saline (PBS) followed by 20 min of postincubation. The digested tissue was triturated to a single-cell suspension by pipetting, and cells were pelleted at 200 × *g* for 5 min. Cells were washed three times in proliferation medium (Neural Stem Cell Basal Medium [SCM003, Merck Millipore] supplemented with B27 without vitamin A [12587010, Thermo Fisher Scientific], GlutaMAX [35050061, Thermo Fisher Scientific], Antibiotic-Antimycotic [15240062, Thermo Fisher Scientific], 1 μg/mL epidermal growth factor [E9644, Sigma-Aldrich], and 1 μg/mL fibroblast growth factor 2 [100-18B, PeproTech]) and plated into single wells of a 24-well plate. Half the proliferation medium was continually changed every other day. Neurospheres formed after approximately 9–12 days.

For passaging, neurospheres were collected and sedimented at 200 × *g* for 5 min. Digestion and trituration to single cells were done in 0.05% trypsin-EDTA (T4174, Sigma-Aldrich), stopped by adding an equal volume of trypsin inhibitor, and cells were sedimented at 200 × *g* for 5 min. Dissociated cells were seeded in proliferation medium at 1 × 10^5^ cells/mL. Neurospheres were passed approximately every third day. All experiments were performed on aNSCs between 3 and 10 passages.

### aNSC Differentiation Assays

aNSCs were seeded on poly-L-ornithine- and laminin-coated (20 μg/mL and 5 μg/mL, respectively) chamber slides in proliferation medium. After 16 h and then every day for 4 consecutive days, half the medium was exchanged with differentiation medium (Neural Stem Cell Basal Medium, supplemented with B27 without vitamin A, GlutaMAX, Antibiotic-Antimycotic, 1 μM forskolin [F6886, Sigma-Aldrich], and 1 μM retinoic acid [R2625, Sigma-Aldrich]). Recombinant mouse RGMa was added to the differentiation medium to give a final concentration of 1 μg/mL. For ROCK inhibition, Y-27632 (688000, Calbiochem) was added to the differentiation medium to give a final concentration of 10 μM.

For siRNA experiments, 16 h after plating, aNSCs were transfected with siRNA using Lipofectamine RNAiMAX Reagent (13,778-100, Invitrogen). siRNA oligos were ordered as annealed double-stranded RNA with a UU overhang (FASMAC) and dissolved in nuclease-free water. Neogenin siRNA sequence: GAA ACA ACC UGC UAA CAU A; NT siRNA sequence: UGU AUU ACG AUU GGU UGU C. After 36 h following transfection and then every day for 4 consecutive days, media were exchange with differentiation media.

On the fifth day (sixth day for GFAP immunocytochemistry) of differentiation, cells were washed with PBS and either lysed directly in the wells for RNA extraction or fixed with 4% paraformaldehyde for 30 min for immunocytochemistry.

### RhoA Assay

RhoA-GTP pull-down was performed with a commercial Rhotekin-RBD kit following the manufacturer's instructions (STA-403-A, Cell Biolabs). In brief, aNSCs were seeded on poly-L-ornithine- and laminin-coated plates in proliferation medium, which after 16 h was exchanged with differentiation medium. Two days later, recombinant mouse RGMa was added (1 μg/mL) and cells were incubated for 1 h at 37°C, followed by PBS wash and cell lysis. Active RhoA-GTP was pulled down from total cell lysate using Rhotekin-RBD agarose beads for 1 h at 4°C. As a negative control, pull-down from total cell lysate was performed together with 100 μM guanosine diphosphate (GDP). After capture the beads were washed three times, and captured proteins were released by boiling the beads in SDS sample buffer for 5 min. Total cell lysate and pull-down lysate was analyzed by western blot for RhoA.

### AAV Plasmid Constructs and Virus Production

Full-length RGMa was amplified from adult hippocampal cDNA and cloned into the pAAV-MCS vector (Stratagene). GFP inserted into the pAAV-MCS vector was used as a control.

shRNA constructs were ordered as single DNA strands (FASMAC), annealed, and ligated into pAAV-H1-shRNA-CMV-tRFP (Yamada et al., 2019). shRNA sequence: shRGMa: CAA CTA CAC TCA CTG CGG CCT; loop sequence: TTC AAG AGA. pAAV-H1-shLuc-CMV-tRFP was used as a nontarget control.

AAV serotype 9 was used in all experimental conditions.

For virus production, AAV HEK293 cells were grown in 10-cm dishes in Dulbecco’s modified Eagle’s medium supplemented with 10% fetal bovine serum. At 80% confluence, pAAV, Rep-Cap plasmid, and helper plasmid were transfected (1:1:2 ratio) using calcium phosphate. Five days later, transfected cells were washed with PBS and collected, and virus particles were extracted using the AAVpro Purification Kit (6666, TaKaRa). Titration was performed using the AAVpro Titration Kit (6233, TaKaRa).

### Stereotaxic Injection of AAV

Mice were anesthetized using a mixture of 0.5 mg/mL butorphanol (Vetorphale, Meiji Seika Pharma), 0.4 mg/mL midazolam (Dormicum, Roche), and 0.03 mg/mL medetomidine (Domitor, Orion Pharma). Using custom pulled glass pipettes, 0.5 μL of AAV particles was injected over 10 min (50 nL/min) at the following coordinates: caudal 2.5 mm, lateral ±1.5 mm, and ventral 1.9 mm from bregma. After the surgery, mice were monitored closely for any complications and used for further experiments 12–14 days after AAV infection.

### Statistics

Statistical analyses and corresponding p values are listed in figure legends and Table S1. Prior to statistical testing, data were tested for normality and model fit by QQ plot and residual plot. Equal variance between groups was tested by homoscedasticity plot or F test. Unpaired Student's t test was used to determine statistical significance for data in Figures 2E–G, 2I, 2K, 3F, 3H–3J, 4B–4E, 4G, 4H, 4J, and 4L. One-way ANOVA followed by Tukey's multiple comparisons test was used to determine statistical significance for data in Figures 1G and 1I–1K. Two-way ANOVA followed by Tukey's multiple comparisons test was used to determine statistical significance for data in Figures 5C, 5D, 5G, and 5H. Two-way ANOVA followed by Sidak's multiple comparisons test was used to determine statistical significance for data in Figure 2M. Statistical significance was defined as p < 0.05.

## Author Contributions

T.J.I., Y.F., and T.Y. designed the experiments. T.J.I. performed the experiments and analyzed the data. T.J.I. wrote the manuscript. T.Y. coordinated and directed the project. All authors discussed the results and commented on the manuscript.

## Supplemental Information

Document S1. Supplemental Experimental Procedures, Figures S1–S3, and Table S1

Document S2. Article plus Supplemental Information
