# Supplementary material for: Repulsive Guidance Molecule A Suppresses Adult Neurogenesis
Source: Stem Cell Reports. 2020 Apr 2;14(4):677–91. doi: 10.1016/j.stemcr.2020.03.003 (PMC7160374; doi:10.1016/j.stemcr.2020.03.003)
Supplement: Document S1. Supplemental Experimental Procedures, Figures S1–S3, and Table S1 [file mmc1.pdf]

Stem Cell Reports, Volume *14*

Supplemental Information

Repulsive Guidance Molecule A Suppresses Adult Neurogenesis

Toke Jost Isaksen, Yuki Fujita, and Toshihide Yamashita

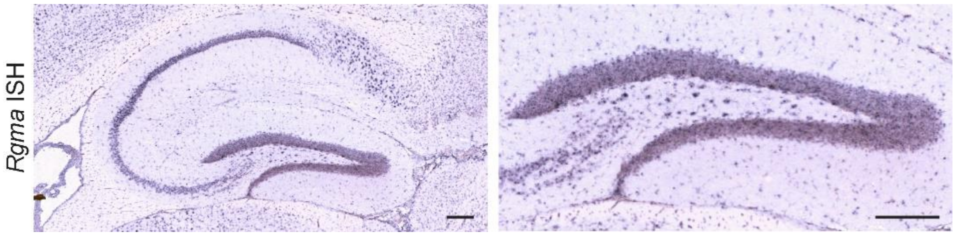

Figure S1. *Rgma* mRNA is expressed in hippocampus, related to Figure 1.
In situ hybridization for *Rgma* mRNA in the hippocampus of an eight-week-old mouse. http://mouse.brain-map.org. Scale bars, 100 µm.

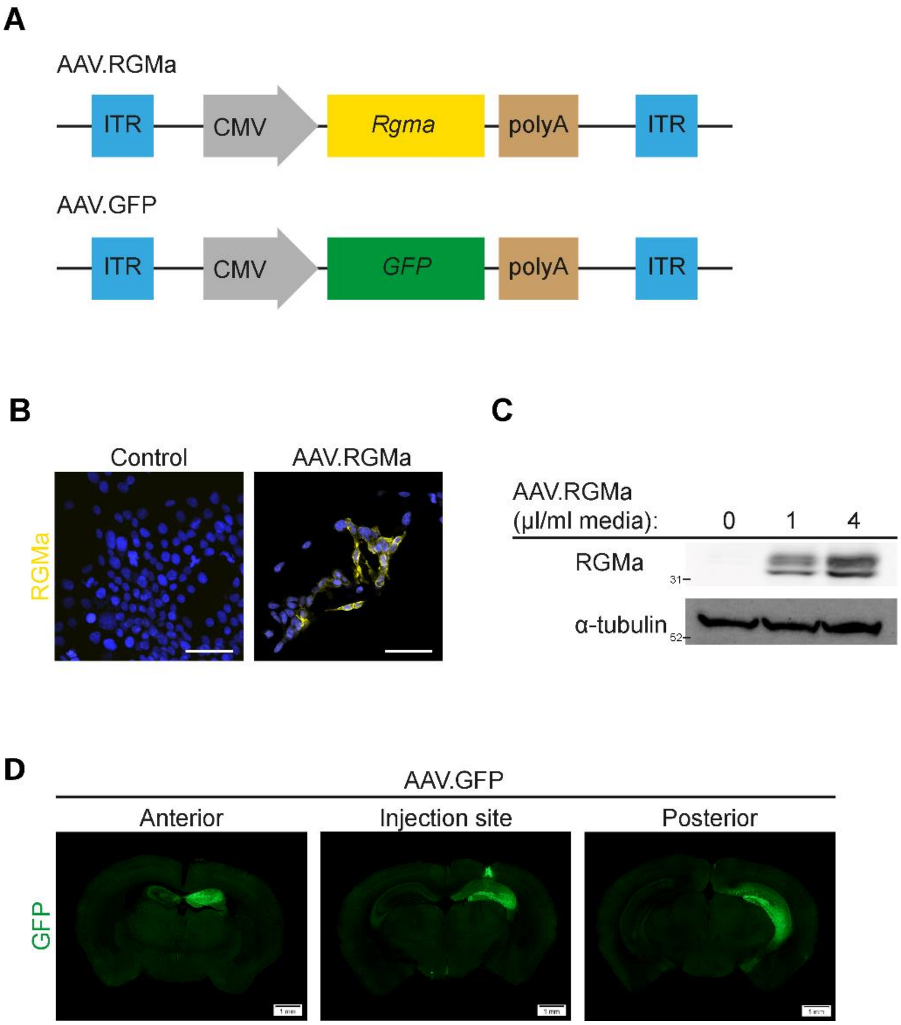

Figure S2. Expression and targeting of RGMa, related to Figure 1.

(A) Full-length RGMa and GFP were cloned into pAAV-MSC for overexpression driven by the CMV promoter.

(B) 293 cells were infected with AAV.RGMa particles and stained for RGMa. Scale bars, 50 µm.

(C) 293 cells were infected with AAV.RGMa particles, and total cell lysate was analyzed by western blot for RGMa.

(D) AAV.GFP particles were injected into the dentate gyrus giving rise to a prominent GFP expression throughout the dentate gyrus formation. Scale bars, 1 mm.

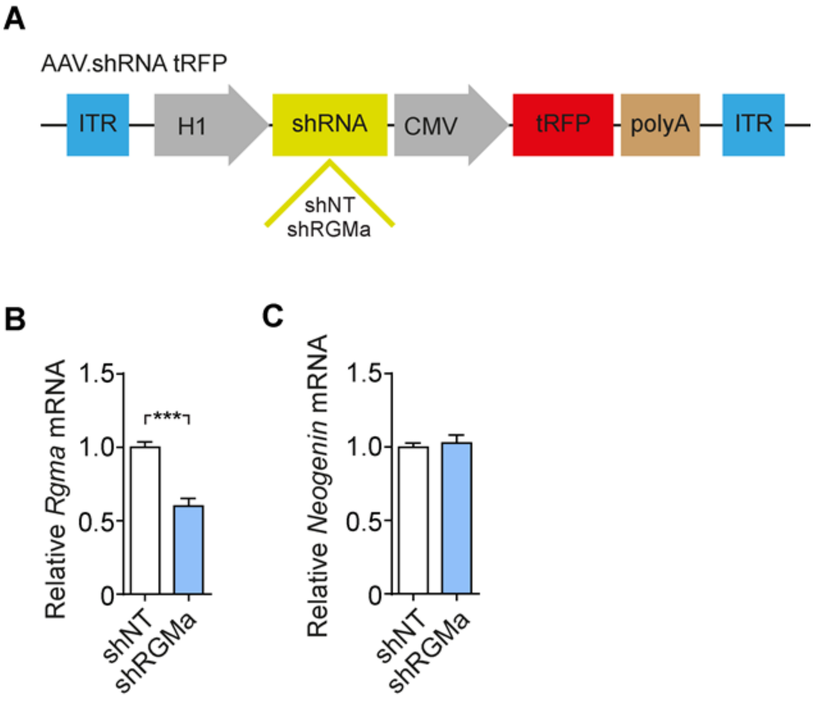

Figure S3. Knockdown of *Rgma* mRNA, related to Figure 2.
(A) AAV.shRNA tRFP constructs.
(B and C) qPCR analysis of *Rgma* (B) and *Neogenin* (C) mRNA levels in the dentate gyrus, two weeks after AAV-infection with shNT and shRGMa (mean ± SEM; n = 7 mice; Unpaired Student’s t-test).

Table S1. Statistical analysis and corresponding p-values, related to all Figures.

Figure	Statistical test	Pairwise comparison	p	Significance
1G	One-way ANOVA, Tukey's multiple comparison test	Control vs AAV.GFP	0.9606	ns
		Control vs AAV.RGMa	0.7633	ns
		AAV.GFP vs AAV.RGMa	0.9059	ns
1I	One-way ANOVA, Tukey's multiple comparison test	Control vs AAV.GFP	0.8537	ns
		Control vs AAV.RGMa	0.0146	*
		AAV.GFP vs AAV.RGMa	0.0273	*
1J	One-way ANOVA, Tukey's multiple comparison test	Control vs AAV.GFP	0.6209	ns
		Control vs AAV.RGMa	0.0013	**
		AAV.GFP vs AAV.RGMa	0.0041	**
1K	One-way ANOVA, Tukey's multiple comparison test	Control vs AAV.GFP	0.9256	ns
		Control vs AAV.RGMa	0.9256	ns
		AAV.GFP vs AAV.RGMa	0.7121	ns
2E	Unpaired t-test	shNT vs shRGMa	0.0369	*
2F	Unpaired t-test	shNT vs shRGMa	0.0133	*
2G	Unpaired t-test	shNT vs shRGMa	0.8194	ns
2I	Unpaired t-test	shNT vs shRGMa	0.7514	ns
2K	Unpaired t-test	shNT vs shRGMa	0.0088	**
2I	Two-way ANOVA, Sidak's multiple comparisons test	shNT vs shRGMa bin -0.5	>0.9999	ns
		shNT vs shRGMa bin -0.4	>0.9999	ns
		shNT vs shRGMa bin -0.3	0.9313	ns
		shNT vs shRGMa bin -0.2	0.0002	***
		shNT vs shRGMa bin -0.1	<0.0001	***
		shNT vs shRGMa bin 0.0	0.9825	ns
		shNT vs shRGMa bin 0.1	>0.9999	ns
		shNT vs shRGMa bin 0.2	>0.9999	ns
		shNT vs shRGMa bin 0.3	0.9998	ns
		shNT vs shRGMa bin 0.4	>0.9999	ns
		shNT vs shRGMa bin 0.5	>0.9999	ns
		shNT vs shRGMa bin 0.6	>0.9999	ns
		shNT vs shRGMa bin 0.7	>0.9999	ns
		shNT vs shRGMa bin 0.8	>0.9999	ns
		shNT vs shRGMa bin 0.9	>0.9999	ns
3F	Unpaired t-test	Control vs RGMa	0.4542	ns
3H	Unpaired t-test	Control vs RGMa	0.7902	ns
3I	Unpaired t-test	Control vs RGMa	0.9862	ns
3J	Unpaired t-test	Control vs RGMa	0.8882	ns
4B	Unpaired t-test	Control vs RGMa	0.0014	**
4C	Unpaired t-test	Control vs RGMa	0.7610	ns
4D	Unpaired t-test	Control vs RGMa	0.0237	*
4E	Unpaired t-test	Control vs RGMa	0.5002	ns
4G	Unpaired t-test	Control vs RGMa	0.0043	**
4H	Unpaired t-test	Control vs RGMa	0.0248	*
4J	Unpaired t-test	Control vs RGMa	0.0005	***
4L	Unpaired t-test	Control vs RGMa	0.0199	*
5C	Two-way ANOVA, Tukey's multiple comparison test	siNT Control vs siNeo Control	0.9613	ns
		siNT Control vs siNT RGMa	0.0005	***
		siNT Control vs siNeo RGMa	0.5028	ns

		siNeo Control vs siNT RGMa	<0.0001	***
		siNeo Control vs siNeo RGMa	0.1210	ns
		siNT RGMa vs siNeo RGMa	0.0236	*
5D	Two-way ANOVA, Tukey's multiple comparison test	siNT Control vs siNeo Control	0.7945	ns
		siNT Control vs siNT RGMa	0.0036	**
		siNT Control vs siNeo RGMa	0.7550	ns
		siNeo Control vs siNT RGMa	0.0277	*
		siNeo Control vs siNeo RGMa	0.9999	ns
		siNT RGMa vs siNeo RGMa	0.0324	*
5G	Two-way ANOVA, Tukey's multiple comparison test	Control vs RGMa	0.0114	*
		Control vs Y27632 Control	0.9823	ns
		Control vs Y27632 RGMa	0.9983	ns
		Y27632 Control vs RGMa	0.0050	**
		RGMa vs Y-27632 RGMa	0.0079	**
		Y27632 Control vs Y27632 RGMa	0.9969	ns
5H	Two-way ANOVA, Tukey's multiple comparison test	Control vs RGMa	<0.0001	***
		Control vs Y27632 Control	0.9731	ns
		Control vs Y27632 RGMa	0.0920	ns
		Y27632 Control vs RGMa	<0.0001	***
		RGMa vs Y-27632 RGMa	<0.0001	***
		Y27632 Control vs Y27632 RGMa	0.0391	*
5J	Two-way ANOVA, Tukey's multiple comparison test	Control vs RGMa	0.0021	**
		Control vs Y27632 Control	0.9557	ns
		Control vs Y27632 RGMa	0.4638	ns
		Y27632 Control vs RGMa	0.0058	**
		RGMa vs Y-27632 RGMa	0.0417	*
		Y27632 Control vs Y27632 RGMa	0.7601	ns
S3A	Unpaired t-test	shNT vs shRGMa	<0.0001	***
S3B	Unpaired t-test	shNT vs shRGMa	0.6483	ns

Supplemental Experimental Procedures

Neurosphere proliferation assay

aNSCs were seeded into 24-well culture plates at 1×10^5 cells/ml in proliferation media supplemented with 1 µg/ml recombinant mouse RGMa. After 48 h, the numbers of formed neurospheres and average neurosphere diameters were assessed in three 2-mm^2 squares in each well. Afterwards, suspended neurospheres were collected, dissociated in 0.05% trypsin-EDTA, and total live cell concentrations were determined using a cell counter.

aNSC BrdU assay

aNSCs were seeded on poly-L-ornithine- and laminin-coated (20 µg/ml, P4957 and 5 µg/ml, L2020, respectively; both Sigma-Aldrich) chamber slides in proliferation media. Two hours after seeding, 1 µg/ml recombinant mouse RGMa was added (1109-N1-025, R&D Systems). After 20 h, dividing cells were labeled with a 10 µM BrdU (B5002, Sigma-Aldrich) pulse for 6 h. Cells were washed with PBS and fixed with 4% paraformaldehyde (PFA) for 30 min. Fixed cells were treated with 1 M HCl for 30 min at 37 °C, followed by neutralization in Borate buffer pH 8.4 for 30 min at room temperature (RT). Blocking was performed in PBS containing 3% bovine serum albumin (BSA) and 0.2% TX-100. Anti-BrdU 1:500 (M0774, Dako) was incubated for 2.5 h at RT or overnight at 4 °C, followed by secondary labeling with Alexa Fluor-conjugated secondary antibodies 1:350 for 1 h at RT. Nuclei were counterstained with 4',6-diamidino-2-phenylindole (DAPI) and coverslips were mounted with Dako fluorescent mounting medium (S3023, Dako). The proliferation rate was calculated by the number of BrdU-positive cells divided by the total number of DAPI-positive cells, both determined using automated threshold particle analysis in ImageJ.

BrdU *in vivo* assay

Mice were injected intraperitoneally with 100 µg/kg BrdU for four consecutive days. On the fifth day or four weeks later, animals were sacrificed, and brains were collected for cryosectioning. Sections spanning the hippocampus were collected and treated with 2 M HCl at 40 °C for 15 min, followed by neutralization with Borate buffer pH 8.4. Sections were blocked for 1 h at RT in PBS containing 0.3% Tx100 and 5.0% BSA. Primary antibodies (anti-BrdU 1:200 [M0774, Dako] and anti-NeuN 1:600 [ABN78, Merck Millipore]) were applied overnight at 4 °C followed by secondary labeling for 1 h at RT using Alexa Fluor-conjugated secondary antibodies 1:350. Nuclei were counterstained with DAPI, and coverslips were mounted with Dako fluorescent mounting medium. Sections evenly spanning ±0.5 mm rostral/caudal from the injection site were analyzed using an FV3000 confocal microscope. The number of BrdU-positive cells in the dentate gyrus was determined by an automated threshold particle analysis in ImageJ. The volume was determined from the outer area of the granular cell layer measured in ImageJ and the section thickness. For migration analysis, the relative migration of each $BrdU^+$ $NeuN^+$ cell into the granular cell layer was calculated as the length from the outer edge of the granular cell layer to the $BrdU^+$ $NeuN^+$ cell (yellow lines in Figure 2H) relative to the length from the outer edge to the inner edge of the granular cell layer (green lines in Figure 2H). A frequency analysis (0.1 bin width) of relative cell migration was calculated for each animal and afterwards averaged for all animals of the same condition.

Immunocytochemistry

For immunocytochemistry, cells were permeabilized in PBS containing 0.2% TX-100 for 15 min and blocked in PBS with 3% BSA for 30 min. Primary antibodies (anti-MAP2 1:500 [M4403, Sigma-Aldrich], anti TuJ1 1:600 [801201, Biolegend], anti-GFAP 1:2 [GA52461-2J, Dako], anti-

cleaved caspase-3 1:400 [9664, Cell Signaling], anti-nestin 1:200 [Mab353, Merck Millipore], or anti-SOx2 1:200 [Mab4343, Merck Millipore]) were applied overnight at 4 °C in block buffer. Secondary labeling was performed using Alexa Fluor-conjugated secondary antibodies 1:350 at RT for 2 h. Nuclei were counterstained with DAPI, and coverslips were mounted with Dako fluorescent mounting medium. Stained cells were analyzed using an FV3000 confocal microscope (Olympus). The ratio of differentiated cells was calculated as the number of MAP2- and GFAP-positive cells (neurons and astrocytes, respectively) divided by the total number of cells stained with DAPI, determined using automated threshold particle analysis in ImageJ. The average MAP2 area per neuron was determined by an automated threshold area analysis of MAP2 in ImageJ divided by the number of MAP2-positive cells. Neurite length was determined by neurite tracing using Simple Neurite Tracer plugin in ImageJ. 15-25 cells for each independent experiment was analyzed. The ratio of apoptotic cells was calculated as the number of caspase-3-positive cells divided by the total number of cells stained with DAPI.

Immunohistochemistry

Cryosections were blocked for 1 h at RT in PBS containing 0.3% TX-100 and 5.0% BSA. Primary antibodies (anti-RGMa 1:200 [28045, Immuno-Biological Laboratories], anti-GAD67 1:400 [Mab5406, Merck Millipore], anti DCX 1:500 [4604, Cell Signaling], anti-SOX2 1:600 [Mab4343, Merck Millipore], or anti-nestin 1:400 [Mab353, Merck Millipore]) was applied overnight at 4 °C followed by secondary labeling for 1 h at RT using Alexa Fluor-conjugated secondary antibodies 1:350 (Invitrogen). Nuclei were counterstained with DAPI, and coverslips were mounted with Dako fluorescent mounting medium. Sections were analyzed using an FV3000 confocal microscope (Olympus). For Sox2 analysis, Sox2 positive cells along the SGZ were counted using

ImageJ cell counter plugin. DCX positive cells were counted using ImageJ cell counter plugin and normalized to the area of the GCL measured in ImageJ.

qPCR and two-step RT-PCR

Total RNA was isolated by RNeasy mini/micro kit (74106/74004, Qiagen) and reverse transcribed into cDNA using the High-Capacity cDNA Reverse Transcription Kit (4368814, Thermo Fisher Scientific). qPCR was performed using Fast SYBR Green Master Mix (4385612, Thermo Fisher Scientific) in a Quant Studio 7 cycler (Applied Biosystems). rTaq polymerase (TAP-201, Toyobo) was used for two-step RT-PCR, and products were analyzed on 2% agarose gels. Primers (Forward, Reverse) were defined as follows: RGMa: CCACATCAGGAAGGCAGAAG, GCGTAGCACTGGGTAGGAAG. GAPDH: TGTGTCCGTCGTGGATCTGA, TTGCTGTTGAAGTCGCAGGAG. Neogenin: TCCAAACACAATAAGCCTGACG, ATGGGACCAAATCTGCATTAACT. Nestin: AGGCTGAGAACTCTCGCTTGC, GGTGCTGGTCCTCTGGTATCC. TuJ1: TATGAAGATGATGACGAGGAATCG, TACAGAGGTGGCTAAAATGGGG. GFAP: CCAAGCCAAACACGAAGCTAA, CATTTGCCGCTCTAGGGACTC.

Western blots

Samples were lysed in 10 mM Tris, 150 mM NaCl, 2 mM EDTA with 1% IGEPAL and Complete protease inhibitor (Roche), separated by SDS-PAGE on 5–10% precast gels (13071-64, Nacalai) or custom-made 10% gels, and electroblotted onto polyvinylidene fluoride membranes (Immobilon-P, Millipore). Membranes were blocked for 1 h at RT in PBS containing 5% skim milk and 0.1% Tween, and incubated with primary antibodies (anti-RGMa 1:1000 [28045, Immuno-Biological Laboratories], anti-RhoA 1:2000 [240302-T, Cell Biolabs], anti-actin 1:2000 [4967S, Cell Signaling Technology], or anti-tubulin 1:2000 [2144, Cell Signaling Technology])

overnight at 4 °C. Secondary labeling was performed using peroxidase-conjugated antibodies (HRP-conjugated anti-mouse IgG [7076, Cell Signaling Technology] or HRP-conjugated anti-rabbit IgG [7074, Cell Signaling Technology]) for 1 h at RT. Membranes were visualized in a ChemiDoc imager (Bio-Rad). For RGMa quantification, the 33 kDa processed C-terminal RGMa form was analyzed by densitometry analysis in ImageJ and normalized to loading control (actin or tubulin) after stripping and reprobing the blot.
